# Comparison Characteristics of Family and Demographic of Children with Antenatal Hydronephrosis between 2^nd^ and 3^rd^ Trimesters of Gestation

**Published:** 2018-02

**Authors:** Maryam NAZEMIPOUR, Abdol-Mohammad KAJBAFZADEH, Kazem MOHAMMAD, Abbas RAHIMI FOROUSHANI, Asal HOJJAT, Maryam SEYEDTABIB, Ali NAZEMIPOUR, Mahmood MAHMOUDI

**Affiliations:** 1. Dept. of Epidemiology and Biostatistics, School of Public Health, International Campus, Tehran University of Medical Sciences, Tehran, Iran; 2. Pediatric Urology Research Center, Dept. of Pediatric Urology, Children’s Hospital Medical Center, Tehran University of Medical Sciences, Tehran, Iran; 3. Dept. of Epidemiology and Biostatistics, School of Public Health, Tehran University of Medical Sciences, Tehran, Iran; 4. Dept. of Epidemiology and Biostatistics, School of Public Health, Hamadan University of Medical Sciences, Hamadan, Iran; 5. Dept. of Computer Engineering, Sharif University of Technology, International Campus, Kish Island, Iran

**Keywords:** Antenatal hydronephrosis, Trimesters of pregnancy, Consanguinity marriage

## Abstract

**Background:**

The aim of this study was comparison characteristics of family and demographics of children with antenatal hydronephrosis, in 2^nd^ and 3^rd^ trimester of pregnancy, in order to the need for postnatal management.

**Methods:**

This cross-sectional study described some information from family of children with antenatal hydronephrosis, at the Pediatric Urology Research Center of Children’s Hospital Medical Center of Tehran University of Medical Sciences. Data for 193 children, admitted in 2012–2013, were collected retrospectively. They were allocated to two groups of 2^nd^ and 3^rd^ trimester, based on the time of diagnosis antenatal hydronephrosis. Data analysis was done using SPSS. Chi-Square, Fisher-exact and independent t-test also Mantel-Hanzel test were used. *P*-value<0.05 was considered as statistically significant.

**Results:**

Of 193 infants (36 female, 157male), the antenatal hydronephrosis of 76 cases (39.4%) have been diagnosed in the 2^nd^ trimester. In addition, 110 cases (57%) were bilateral and 33 cases (17.1%) had severe antenatal hydronephrosis. Consanguinity marriage, being unilateral or bilateral and the severity of antenatal hydronephrosis were significant with the specific trimester that it has been diagnosed (*P*<0.05).

**Conclusion:**

Infants with bilateral and severe grade of antenatal hydronephrosis also with the history of consanguinity marriage among their parent will diagnose in the second trimester more than the third trimester and will refer for some surgical correction, relating to other kidney diseases such as vesicoureteral reflux, more than others. Thus, this is a good sign for caring infants.

## Introduction

Of genitourinary anomalies, which make up approximately 20% of all antenatal detected fetal anomalies (1), hydronephrosis is one of the most common abnormality observed around 1% to 5% of all pregnancies. There are various causes that trigger its incidence (2–7).

The ureteric bud, the metanephric blastema, and the cloaca are three interrelated separate structures that make up the kidney. The excretory system, including the ureter, renal pelvis, calyxes, papillary and collecting duct, are completely formed by the 20^th^ weeks of pregnancy (8).

Since the anomalies of the urinary tract are progressive, besides performing fetal ultrasound for the obstetric management before the 24^th^ wk of gestation, maternal-fetal ultrasound should be performed after the 28^th^ wk of pregnancy too, in order to detect other alterations of the urinary tract that were not detectable between the 20^th^ and the 24^th^ wk (9, 10).

Antenatal hydronephrosis (ANH) as distention of renal calyces and pelvis or dilatation of renal collecting system, which is detected frequently by maternal-fetal ultrasound and before birth, is one of the alterations of the urinary tract (11–13).

The need to act fast after detecting hydronephrosis during any period of pregnancy may depend on the degree of dilatation and be unilateral or bilateral too.

In general, accurately classifying the degree of upper urinary tract dilatation in a fetus or neonate is difficult. Therefore, there are inconsistencies in presenting the degree of ANH in different researchers’ reports, which reflects a lack of consensus on ANH grading (14). Many studies have reported the anterior-posterior diameter of the renal pelvis (APD) for the fetal as a standard classification of ANH. In 1990, Mandell proposed a classification system based on the APD and helps to categorize ANH in the mild, moderate and severe (15, 16). Later, in a large meta-analysis, ANH was classified into five groups, based on the measures of the APD in second and third trimester of gestation (17). However, employing three groups of mild, moderate and severe based on the APD in the second and third trimester, is in line with the majority of studies undertaken so far (18, 19).

There is only a small amount of evidence based on data about postnatal evaluation of the fetal hydronephrosis. Therefore, the aim of this study was comparison of characteristics of family and demographic of children with ANH in 2^nd^ and 3^rd^ trimester of gestation, in order to the need for postnatal management.

## Materials and Methods

This cross-sectional study was carried out to describe and collect some information retrospectively from family of children with ANH at the Pediatric Urology Research Center of Children’s Hospital Medical Center of Tehran University of Medical Sciences, Tehran, between 2012–2013.

ANH for almost 40% of the children was diagnosed in 2^nd^ trimester of pregnancy. Therefore, required number of children with ANH, by using formula n=z1−α22p(1−p)d2 and considering 95% for level of confidence with tolerated error less than 7%, will be 193 as a sample size for this study. Thus, using the model of Poisson sampling method, 193 children with ANH admitted in the center between 2012–2013 were enrolled in this study.

All the data were collected after Tehran University of Medical Sciences Institutional Review Board (TUMS IRB) approval.

The eligibility criteria included children with a definitive diagnosis of ANH admitted to the center from 2012–2013. Some information related to their demographic, clinical and family characteristics was collected from their medical records and by phoning their family. In addition, mother’s permission was obtained through this way.

For comparing characteristics of children with ANH in 2^nd^ and 3^rd^ trimester of gestation, children were allocated to two groups, based on the time of diagnosis ANH, in second and third trimester.

The ANH for the children was diagnosed with maternal-fetal ultrasound. The severity of graded based on the measure of the APD at gestational age or specific trimester, and classified into three groups of mild, moderate and severe (7, 20–22). For proper APD calculations, measurements should be obtained from a transverse axial image of the renal pelvis at the approximate level of the renal hilum (16).

In this way, 76 (39.4%) of the ANH cases were diagnosed in the second trimester and 117 (60.6%) of them in the third trimester of pregnancy. Data analysis was done using SPSS (Ver. 22, Chicago, IL, USA). Chi-Square, Fisher-exact test, and independent t-test also Mantel-Hanzel test was used for this paper. *P*-value<0.05 was considered as statistically significant.

## Results

Overall, 76 cases (39.4%) were in the 2^nd^ trimester so that, 64 (84.2%) of them were boy, and 117 cases (60.6%) were in the 3^rd^ trimester so that, 93 (79.5%) of them were boy. The status and association of other variables with the gestational age or specific trimester that ANH has been diagnosed (Table 1).

**Table 1:** Frequency distribution of the children with ANH, according to the 2^nd^ and 3^rd^ trimester of pregnancy, with the other characteristics of them or their family

***Characteristics***	***Status***	***2^nd^ trimester No (%)***	***3^rd^ trimester No (%)***	***Total No (%)***	***P-value***
Gender	Girl	12(15.8)	24(20.5)	36(18.7)	0.265
	Boy	64(84.2)	93(79.5)	157(81.3)	
History of abortion	No	58(76.3)	81(69.2)	139(72)	0.182
	Yes	18(23.7)	36(30.8)	54(28)	
Number of deliveries	None	40(52.6)	67(57.3)	107(55.4)	0.314
	One and more	36(47.4)	50(42.7)	86(44.6)	
Mother’s education	Academic degrees	64(84.2)	100(85.5)	164(85)	0.483
	None	12(15.8)	17(14.5)	29(15)	
Consanguinity marriage	NO	50(65.8)	95(81.2)	145(75.1)	0.013
	YES	26(34.2)	22(18.8)	48(24.9)	
Existence of other kidney diseases besides ANH and VUR	NO	34(44.7)	43(36.8)	77(39.9)	0.169
	YES	42(55.3)	74(63.2)	116(60.1)	
Kidney disease background in parent	NO	72(94.7)	110(94)	182(94.3)	0.55
	YES	4(5.3)	7(6)	11(5.7)	
Abortion	NO	58(76.3)	81(69.2)	139(72)	0.182
	YES	18(23.7)	36(30.8)	54(28)	
Type of treatment	Medicine	26(34.2)	46(39.3)	72(37.3)	0.287
	Surgery	50(65.8)	71(60.7)	121(62.7)	
Mother’s age of marriage (yr) *		22.26(4.69)	22.92(4.63)	22.66(4.65)	0.33
Mother’s age at delivery (yr)*		29.3(4.99)	29.3(5.61)	29.3(5.36)	0.99
Birth spacing (yr) *		5.51(3.59)	5.12(3.16)	5.27(3.33)	0.42
Child’s birthweight (kg)*		3.22(0.5)	3.28(0.43)	3.26(0.46)	0.35
Grade Of ANH	Mild	52(68.4)	82(70.1)	134(69.4)	<0.001
	Moderate	5(6.6)	21(17.9)	26(13.5)	
	Severe	19(25)	14(12)	33(17.1)	
Direction Of ANH	Left sided	14(18.4)	38(32.5)	52(26.9)	0.001
	Right sided	7(9.2)	24(20.5)	31(16.1)	
	Bilateral	55(72.4)	55(47)	110(57)	
Total		76(100)	117(100)	193(100)	

* mean (SD)

They are not significant except variables, “Con-sanguinity marriage” (*χ*^2^ = 5.853, *df* = 1, *P* = 0.013), “Grade of ANH” (*χ*^2^ = 9.017, *df* = 2, *P* < 0.001) and “Direction of ANH” (*χ*^2^ = 12.00, *df* = 2, *P* = 0.001), which specifies the involved side of kidney with the ANH (unilateral (left or right side of kidney) or bilateral (both sides)). It means more proportion of children whose ANH have been diagnosed in the 2^nd^ trimester have consanguinity marriage among their parents (34.2% vs 18.8%), severe grade of ANH (25% vs 12%). Moreover, their ANH are bilateral (72.4% vs 47%) in comparison with the children whose ANH have been diagnosed in the 3^rd^ trimester. Mother’s mean age of marriage in the two groups of the second and third trimester was 22.26 and 22.92 yr old respectively. Other variables such as, “Mother’s age at delivery date”, “Birth spacing” and “Child’s birthweight” were not significant between the two groups. Therefore, age of the mother, birth interval of the child with his previous sibling and child’s birthweight are not associated with the time of diagnosing ANH.

Table 2 displays the frequency distribution of the children, according to their grade of ANH and their type of treatment, in the 2^nd^ and 3^rd^ trimester. There is no significant association between the type of treatment and the trimester which ANH has been diagnosed, neither in each grade of the ANH nor in total or even with adjusting for the effect of the variable “Grade of ANH” ( $\chi_{\text{MH}}^{2}=0.032$ , *P*=0.858).

**Table 2:** Frequency distribution of the children with ANH, according to their grade of ANH and their type of treatment, in the 2^nd^ and 3^rd^ trimester of pregnancy

***Grade of ANH***	***2^nd^ trimester No (%)***		***3^rd^ trimester No (%)***		***Total No (%)***		***P-value***
	**Type of Treatment**		**Type of Treatment**		**Type of Treatment**		
	**Medicine**	**Surgery**	**Medicine**	**Surgery**	**Medicine**	**Surgery**	
Mild	18(34.6)	34(65.4)	28(34.1)	54(65.9)	46(34.3)	88(65.7)	0.55
Moderate	3(60)	2(40)	12(57.1)	9(42.9)	15(57.7)	11(42.3)	0.654
Severe	5(26.3)	14(73.7)	6(42.9)	8(57.1)	11(33.3)	22(66.7)	0.266
Total	26(34.2)	50(65.8)	46(39.3)	71(60.7)	72(37.3)	121(62.7)	0.287

Sample size in each group is very small and that is why, except for the first level of grade, which is in borderline, there is no significant association between the consanguinity marriage and trimester in different grade of the ANH. However, it is statistically significant (*P*-value=0.013). In addition, $\chi_{\mathrm{MH}}^{2}$ shows by adjusting for the effect of the grade of ANH, it is seen a significant association between those variables ( $\chi_{\mathrm{MH}}^{2}=4.524$ , *P*=0.033) (Table 3).

**Table 3:** Frequency distribution of the children with ANH, according to their grade of ANH and status of consanguinity marriage among their parents, in the 2^nd^ and 3^rd^ trimester of pregnancy

***Grade of ANH***	***2^nd^ trimester No (%)***		***3^rd^ trimester No (%)***		***Total No (%)***		***P-value***
	***Consanguinity marriage***		***Consanguinity marriage***		***Consanguinity marriage***		
	***NO***	***YES***	***NO***	***YES***	***NO***	***YES***	
Mild	34(65.4)	18(34.6)	64(78)	18(22)	98(73.1)	36(26.9)	0.08
Moderate	3(60)	2(40)	19(90.5)	2(9.5)	22(84.6)	4(15.4)	0.155
Severe	13(68.4)	6(31.6)	12(85.7)	2(14.3)	25(75.8)	8(24.2)	0.234
Total	50(65.8)	26(34.2)	95(81.2)	22(18.8)	145(75.1)	48(24.9)	0.013

It was not seen any significant association between variable “type of treatment” and the trimester, neither in the level of the “Direction of ANH”, nor in total or even with adjusting for the effect of this variable ( $\chi_{\mathrm{MH}}^{2}=0.058$ , *P*=0.81) (Table 4). There is a significant association between the variable “consanguinity marriage” and the trimester, being just in the bilateral level of ANH. Like in the Table 3, the sample size was small (Table 5). Therefore, it was not seen any significant association for other levels of this variable. However, by removing the effect of the “Direction of ANH”, it is still significant ( $\chi_{\mathrm{MH}}^{2}=4.873$ , *P*=0.027).

**Table 4:** Frequency distribution of the children with ANH, according to the involved side with the ANH and their type of treatment, in the 2^nd^ and 3^rd^ trimester of pregnancy

***Direction of ANH***	***2^nd^ trimester No (%)***		***3^rd^ trimester No (%)***		***Total No (%)***		***P-value***
	***Type of treatment***		***Type of treatment***		***Type of treatment***		
	***Medicine***	***Surgery***	***Medicine***	***Surgery***	***Medicine***	***Surgery***	
Left sided	7(50)	7(50)	19(50)	19(50)	26(50)	26(50)	0.622
Right sided	2(28.6)	5(71.4)	8(33.3)	16(66.7)	10(32.3)	21(67.7)	0.599
Bilateral	17(30.9)	38(69.1)	19(34.5)	36(65.5)	36(32.7)	74(67.3)	0.42
Total	26(34.2)	50(65.8)	46(39.3)	71(60.7)	72(37.3)	121(62.7)	0.287

**Table 5:** Frequency distribution of the children with ANH, according to the involved side with the ANH and status of consanguinity marriage among their parents, in the 2^nd^ and 3^rd^ trimester of pregnancy

***Direction of ANH***	***2^nd^ trimester No (%)***		***3^rd^ trimester No (%)***		***Total No (%)***		***P-value***
	**Consanguinity marriage**		**Consanguinity marriage**		**Consanguinity marriage**		
	**NO**	**YES**	**NO**	**YES**	**NO**	**YES**	
Left sided	9(64.3)	5(35.7)	27(71.1)	11(28.9)	36(69.2)	16(30.8)	0.44
Right sided	6(85.7)	1(14.3)	21(87.5)	3(12.5)	27(87.1)	4(12.9)	0.662
Bilateral	35(63.6)	20(36.4)	47(85.5)	8(14.5)	82(74.5)	28(25.5)	0.008
Total	50(65.8)	26(34.2)	95(81.2)	22(18.8)	145(75.1)	48(24.9)	0.013

## Discussion

Among genitourinary anomalies, hydronephrosis is one of the most common abnormality observed around 1% to 5% of all pregnancies and happens due to various causes (2–7). Parents of fetus detected with antenatal hydronephrosis are interested to know whether the postnatal management is necessary for their children or not. However, correct and proper classifying the degree of upper urinary tract dilatation in a fetus or neonate is still a controversial issue, caused inconsistencies in presenting the degree of ANH, which is important in scrutinizing the patient conditions. Employing three groups of mild, moderate and severe for the severity of ANH, based on the APD in the 2^nd^ and 3^rd^ trimester, is in line with the majority of studies (18, 19).

This study is important as it was designed to determine the characteristics of family and demographic of children with ANH in 2^nd^ and 3^rd^ trimester of gestation, in order to the need for postnatal management. Children with mild and moderate grade of ANH diagnosed in the 2^nd^ trimester and with the history of consanguinity marriage, can be discharged early. Children with severe grade of ANH and with the consanguinity marriage need to follow up by a multi-disciplinary team. It is suggested that fetuses diagnosed with bilateral hydronephrosis be monitored frequently. The frequency of monitoring depends on the gestation at which ANH was detected also its severity (23). However it is said almost 80% of fetuses diagnosed in the 2^nd^ trimester show improvement of findings (6, 22, 24) with low likelihood of postnatal sequelae (24, 25).

The result obtained in this study shows that infants with the bilateral and severe grade of ANH also with the history of consanguinity marriage among their parents, will be diagnosed in the 2^nd^ trimester more than the 3^rd^ one. About 88% cases with mild ANH resolved in embryonic course or neonatal period, while almost 33% neonates with moderate or severe hydronephrosis persisting in the 3^rd^ trimester required postnatal surgery (6). Risk of deterioration is higher for bilateral than for unilateral (26). Patients with mild to moderate isolated bilateral hydronephrosis have a favorable outcome (27), it is necessary to follow up them closely since a proportion may show progression or require surgery (23, 28).

## Conclusion

Results stress the necessity of attention to the infants with the bilateral and severe grade of ANH also with the history of consanguinity marriage among their parents.

## Ethical considerations

Ethical issues (Including plagiarism, informed consent, misconduct, data fabrication and/or falsification, double publication and/or submission, redundancy, etc.) have been completely observed by the authors.
